# Changes in hair cortisol in a New Zealand community sample during the Covid-19 pandemic

**DOI:** 10.1016/j.cpnec.2024.100228

**Published:** 2024-02-15

**Authors:** Elizabeth Broadbent, Urs Nater, Nadine Skoluda, Norina Gasteiger, Ru Jia, Trudie Chalder, Mikaela Law, Kavita Vedhara

**Affiliations:** a Department of Psychological Medicine, University of Auckland, Private Bag 92019, Auckland, New Zealand; b University Research Platform “The Stress of Life (SOLE) – Processes and Mechanisms Underlying Everyday Life Stress”, Department of Clinical and Health Psychology, University of Vienna, Liebiggasse 5, 1010, Vienna, Austria; c School of Health Sciences, University of Manchester, Oxford Road, Manchester, M13 9PL, UK; d Centre for Academic Primary Care, School of Medicine, University of Nottingham, UK; e Department of Psychological Medicine, Institute of Psychiatry, Psychology & Neuroscience, King's College London, 16, De Crespigny Park, London, SE5 8AF, UK; f School of Psychology, Cardiff University, Tower Building, Cardiff, CF10 3AT, UK

**Keywords:** Covid-19, Cortisol, Stress

## Abstract

**Background:**

Evidence suggests that countries with higher Covid-19 infection rates experienced poorer mental health. This study examined whether hair cortisol reduced over time in New Zealand, a country that managed to eliminate the virus in the first year of the pandemic due to an initial strict lockdown.

**Methods:**

A longitudinal cohort study assessed self-reported stress, anxiety and depression and collected hair samples that were analyzed for cortisol, across two waves in 2020. The sample consisted of 44 adults who each returned two 3 cm hair samples and completed self-reports. Hair cortisol was assessed per centimetre.

**Results:**

Hair cortisol reduced over time (F (5, 99.126) = 10.15, p < .001, partial eta squared = 0.19), as did anxiety and depression. Higher hair cortisol was significantly associated with more negative life events reported at wave two (r = 0.30 segment 1, r = 0.34 segment 2, p < .05), but not anxiety or depression.

**Conclusions:**

Strict virus control measures may not only reduce infection rates, but also reduce psychological distress, and hair cortisol over time.

## Highlights

• A hard and fast government response eliminated the Covid-19 virus in New Zealand.
• Over the next three months, hair cortisol, anxiety and depression reduced.
• Negative life events were related to higher hair cortisol.

## 1 Introduction

The Covid-19 pandemic and the lockdowns that were introduced to slow infection rates caused significant disruption to people's lives. Research has demonstrated increased depression, anxiety, and stress in public samples across the world during the pandemic compared to before the pandemic [1].

Differences in the management and course of the pandemic between countries resulted in different infection rates. For example, the United Kingdom (UK) had a slow and inconsistent response, which failed to contain the spread of the virus [2]. On the other hand, New Zealand (NZ) had a hard and fast public health response, resulting in elimination of the virus in the community [3]. Death rates per 100 000 people were 325 in the UK, and 53 in New Zealand [4].

There is evidence that people in countries with higher infection rates experienced higher anxiety and depression than people in countries with lower infection rates, but mixed evidence for effects of levels of the stringency of government response on mental health [5]. This is supported by research showing that although people from both the UK and 10.13039/501100001562NZ reported elevated stress, anxiety, and depression, in the pandemic compared to pre-population norms, stress and anxiety were significantly worse in the UK than in 10.13039/501100001562NZ [6,7].

The measurement of hair cortisol offers a psychobiological marker of changes in stress over time. Hair cortisone was shown to increase during the pandemic in a UK community sample [8]. Similarly, hair cortisol increased in a sample of youths in the USA [9]. However, a community sample from Austria, Germany, and Italy, found lower hair cortisol during lockdown compared to before lockdown, possibly due to lower daily hassles [10].

To further examine the effects of management of the pandemic on hair cortisol, this paper reports changes in hair cortisol over time a NZ sample. Given the rapid stringent response and subsequent community elimination of the virus in NZ, it was hypothesized that hair cortisol would reduce over the period of the study. It was also hypothesized that higher self-reported stress would be associated with higher hair cortisol within the sample.

## 2 Materials and methods

In May 2020, a longitudinal study was commenced in New Zealand to assess the effects of the pandemic on mental health. Ethics approval was obtained from the Auckland Health Research Ethics Committee and all participants gave informed consent. The study was conducted in accordance with the Declaration of Helsinki.

Wave 1 of data collection in NZ was from May 8, 2020 to June 6, 2020, wave 2 was 12 weeks later (July 29, 2020 to September 3, 2020), and wave 3 was more than six months later (March 8, 2021 to April 10, 2021). Self-reported stress, anxiety, and depression were measured across all three waves using the 4-item Perceived Stress Scale (PSS-4 [11]), Generalized Anxiety Disorder-7 (GAD-7 [12]), Patient Health Questionnaire-9 (PHQ-9 [13]). Negative life events from Covid-19 from a checklist were scored as present (1) or absent (0) and summed [14]. The negative life events were death of a close relative/friend, major health event requiring hospitalization for self or loved one, self or partner losing job, change in financial status for the worse, change in living conditions for the worse, and change in personal relations for the worse. More information about the survey and methods is available in earlier papers [7,14].

Participants could volunteer to collect hair samples at the first and second waves of the study, wrap them in foil, label the root end and add the date the samples were taken, and post these to the study centre. The return of hair samples was optional for cultural reasons, respecting the sacred significance that Māori communities traditionally attribute to the head (and hair). The received hair samples were labelled from 4th May to 6th June (wave 1), and 31st July to September 25, 2020 (wave 2). Each 3 cm hair sample was cut into three 1 cm length segments starting from the hair root (corresponding to one month for each segment and a total of three months) and analyzed for hair cortisol concentration (HCC) according to the laboratory protocol described by Feneberg et al. [10]. For cortisol determination, a commercially available cortisol luminescence immunoassay was used (LIA; IBL International, a Tecan Group company, Hamburg, Germany). Inter- and intra-assay coefficients of variation were 2.1% and 7.5%, respectively.

**Fig. 1:**
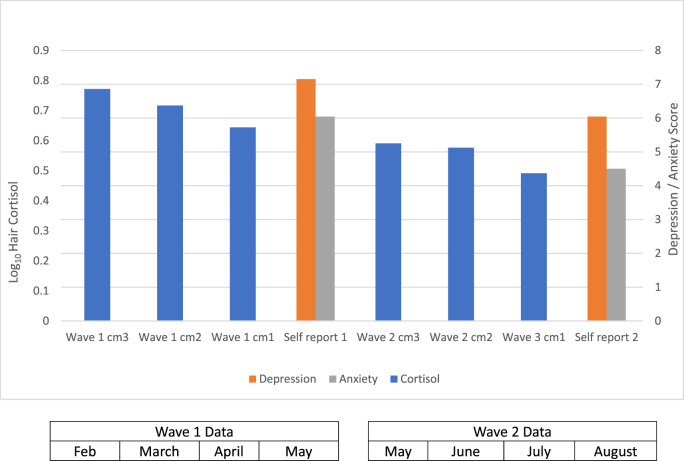
Mean Log_10_ hair cortisol levels reduced across the two waves of the study in 2020. Each 3 cm hair sample was cut into three 1 cm segments for analysis. Mean self-reported anxiety and depression also reduce over time as shown. A time scale is indicated for months for each wave.

Cortisol data were log_10_ transformed to meet the normality assumptions of parametric tests. SPSS was used to analyze data. Pearson correlations were conducted between log_10_ hair cortisol for each 1 cm segment as well as averaged across both 3 cm segments with age, stress, anxiety, depression, and negative life events. Hair segment cm 1 represents cortisol over the month prior to self-reported stress collection, segment cm 2 represents cortisol one to two months prior to self-reported stress, and cm 3 corresponded to cortisol between two-three months prior to self-reported stress (see Fig. 1).

Independent samples t-tests were conducted to test differences between keyworkers and non-keyworkers. Keyworkers were people who continued their work during the social restriction waves of the pandemic due to the essential nature of their professions. Changes in hair cortisol over time were analyzed with repeated measures ANOVA with the Greenhouse Geisser correction due to violation of sphericity. The original power analysis was conducted for self-reported outcomes [7]. For hair cortisol, post-hoc analysis of achieved power for repeated measures ANOVA of six 1 cm hair cortisol segments with effect size f = 0.48 (see results section below) showed that power above .90 was achieved. A p value of .05 was maintained for statistical significance.

## 3 Results

681 participants completed the wave 1 survey, and 375 completed the wave 2 survey [14]. For this paper, only the subset of 44 participants who returned 3 cm hair samples from both waves 1 and 2 were included (12%; 42 females and 2 males). Participants who provided hair samples were significantly older (mean 50.05, SD 13.76) compared to those who completed the surveys but did not return the hair samples (mean 42.58, SD 16.51), t (373) = 2.87, p = .004, but gender and ethnicity proportions did not significantly differ. There were no significant differences in wave 1 depression, anxiety, or stress in those who returned the hair samples at the two time points (mean depression 7.61, SD 6.65; anxiety 6.18, SD 4.75; and stress 5.45, SD 3.53) compared to those who did not (mean depression 7.83, SD 6.46; anxiety 6.19, SD 5.44; and stress 6.25, SD 3.34).

Age was not significantly correlated with hair cortisol. 18/44 (41%) of these participants were healthcare keyworkers, 5/44 were non-health related keyworkers (11%), and 21/44 (48%) were not keyworkers. There was no difference in hair cortisol between keyworkers and others.

Hair cortisol levels significantly reduced in a linear fashion over time using the six 1 cm hair length, (F (5, 99.126) = 10.15, p < .001, partial eta squared = 0.19; Fig. 1).

The mean number of reported negative life events was 0.45 (SD 0.66) at wave 1, and 0.45 (SD 0.63) at time 2. Total negative life events reported at wave 2 were significantly correlated with mean hair cortisol for the 3 cm hair segments at both wave 1 and wave 2. When cortisol in each 1 cm segment was examined separately, correlations with negative life events at wave 2, were significant for wave 1 segment 3 (r = 0.33, p ≤ .029), wave 2 segment 2 (r = 0.32, p = .037, and wave 2 segment 3 (r = 0.34, p = .024). All other correlations with perceived stress, anxiety, and depression, were non-significant (see Table 1).

**Table 1:** Correlations between hair cortisol, stress, and mood measures at both waves.

	Log 10 cortisol 3 cm segment Wave 1	Log 10 cortisol 3 cm segment Wave 2
**Wave 1**		
Perceived Stress	−.06	.12
Depression	.01	.21
Negative life events	.04	.20
Anxiety	.04	.13
**Wave 2**		
Perceived Stress	−.07	.12
Depression	−.04	.17
Negative life events	.30^a^	.34^a^
Anxiety	−.03	.08

a Correlation is significant at the 0.05 level (2-tailed).

## 4 Discussion

There was a linear reduction in hair cortisol over time during the first six months of the pandemic in this New Zealand sample. This stands in contrast to the UK and US studies, where hair cortisone/cortisol increased over a similar period [8,9].

Analysis has shown that countries with lower infection rates experienced lower anxiety and depression [5]. Our results suggest this may also extend to lower hair cortisol. In this study, reductions in hair cortisol were echoed by reductions in self-reported anxiety and depression over this period, and the mean number of reported negative events was low. The reduction in cortisol may have long-term protective effects, as cortisol can affect immune function and adversely affect health outcomes.

There was an association between negative life events reported in the second wave of the study and higher hair cortisol in the preceding months. These results are in line with results from the UK, where higher perceived stress was associated with higher hair cortisone, a corticosteroid metabolite [8].

Limitations of this research include the small number of participants who provided hair samples at both waves, and these participants were significantly older than those who did not provide samples. The low response rate and small sample size mean that the results may not generalize to others outside this sample. Most of the sample were women so results may not generalize to other genders. The small sample size limited power to find significant differences between keyworkers and non-keyworkers. Younger adults were disproportionately affected during the pandemic, and results may not generalize to this group. Other reasons for the reduction in cortisol may be due to seasonal differences (NZ was moving into winter), as summer season has been associated with higher hair cortisol concentrations (Fischer et al., 2016).

## 5 Conclusion

Hair cortisol levels of this small sample in NZ reduced over the first six months of the pandemic. This may be because strict lockdown measures were introduced early in the pandemic resulting in elimination of the virus, and reduced anxiety and depression in this sample.

## Ethical approval

This work was conducted in accordance with the Declaration of Helsinki. Ethical approval was granted from the Auckland 10.13039/100005622Health Research Ethics Committee (Ref: AH1326).

## Funding

The 10.13039/501100001562NZ based research did not receive any specific grant from funding agencies in the public, commercial, or not-for-profit sectors.

[15].

## CRediT authorship contribution statement

**Elizabeth Broadbent:** Writing – review & editing, Writing – original draft, Methodology, Investigation, Formal analysis, Data curation, Conceptualization. **Urs Nater:** Writing – review & editing, Supervision, Methodology. **Nadine Skoluda:** Writing – review & editing, Methodology. **Norina Gasteiger:** Writing – review & editing, Methodology, Data curation, Conceptualization. **Ru Jia:** Writing – review & editing, Methodology, Conceptualization. **Trudie Chalder:** Writing – review & editing, Methodology, Conceptualization. **Mikaela Law:** Writing – review & editing, Data curation. **Kavita Vedhara:** Writing – review & editing, Methodology, Conceptualization.

## Declaration of competing interest

TC is part-funded by the 10.13039/501100000272National Institute for Health Research (10.13039/501100000272NIHR) Biomedical Research Centre at 10.13039/100009362South London and Maudsley NHS Foundation Trust, King's College London. She has grants from Guy's St Thomas Charity Grants, 10.13039/501100000272NIHR and 10.13039/100014013UKRI for post COVID syndromes.

She has received travel expenses, accommodation costs, and honorarium for several lectures in Europe and has received travel expenses and accommodation costs for attending American Thoracic Society Conference. She was on the Expert Advisory Panel for Covid-19 Rapid Guidelines.
